# Provision of Convenient Play Space in a Densely Populated City

**DOI:** 10.3390/ijerph16040651

**Published:** 2019-02-22

**Authors:** Poh-Chin Lai, Chien-Tat Low

**Affiliations:** Department of Geography, The University of Hong Kong, Hong Kong, China; pclai@hku.hk

**Keywords:** play space, children, city, public space, urban environment, Hong Kong

## Abstract

The focus of this study is on examining sufficiency and quality of play space in a densely populated city from the spatial perspective. The study employed a three-stage multilevel mixed-method design using spatial analysis, user questionnaire, and site surveys. Provision of play space was assessed based on location, user perception, and proximity to residential areas and roads. The spatial distribution of play space was mapped and examined by applying GIS-based multicriteria analysis. Without considering play space provided by private housing estates, the study found a mismatch between children population and location of play space. The study also identified stair, slope, and sidewalk conditions as key issues of accessibility to selected playgrounds, even in districts with sufficient play space. Kowloon has limited play space of which a high percentage is inferior in terms of safety and pollution standards. Spatial analysis can help inform optimal locations for play space. Future studies should be based on more well-rounded and complete data to advise urban planning. Additionally, policy makers should focus more on quality standards of play space (i.e., openness, absence of pollution, attraction, safety, etc.) when planning as opposed to simply meeting the minimum area per person quota for open space.

## 1. Introduction

Article 31 of the UN Convention on the Rights of the Child in 1989 [1] states that “every child has the right to rest and leisure, to engage in play and recreational activities appropriate to the age of the child and to participate freely in cultural life and the arts”. Play is essential in promoting interaction and cultivating social values that influence the shaping of society. Having a well-designed play environment for the age range is important in engaging children and youngsters in active sports and recreational activities [2,3]. Play spaces can be outdoor or indoor. They are further characterized according to ease of access, level of supervision, provision of play equipment, age specific activities, landscaping, and inclusivity. The following definition of place space is adapted from UK and US experiences:

A comprehensive definition of play space refers to permanent structures for sports and recreational activities by children. These structures can be staffed or unstaffed, indoor or outdoor, and in public or private grounds. The structures can have formal play equipment or non-equipped areas such as landscaped areas and playing fields that allow for a variety of recreational and physical activities. Some examples include public play areas or playgrounds in parks, basketball courts, football pitches, kickabout areas, multi-use game areas, as well as schools, nurseries, and other educational settings that provide space for physical exercise.

It is believed that children with and without special needs will learn to have comfortable social interactions with one another through playful interaction. To ensure that children’s right to play is not an extra luxury subsequent to considerations of other rights, play space design must be an essential component in the urban planning process [4]. A clear understanding of the current provision of play space (including quantity, quality, and spatial distribution) and potential sites in consideration of various selection criteria (such as transport connectivity, ecological balance, equality and inclusion, usability, and sustainability) is key to proactive planning of future play space. Studies have shown that children prefer play environments with landscaped nature over non-vegetated and artificial settings [5,6,7]. Whereas adults and adolescents make more frequent visits to outdoor public places with trees and vegetation, these more natural environmental settings are also found to support children’s imaginative play and the development of positive relationships [8,9]. It has thus been proposed that early childhood centers and primary schools should have close access to natural outdoor recreational spaces to enhance learning and social intervention [10]. There are no universally accepted levels of physical activity among young children [11]. The US National Recreation and Park Association recommends 120 min of physical activity per day although the levels do vary between boys and girls in different physical activity context and degrees of independent mobility [12]. 

Before the provision of public play spaces in a built environment, children played on the streets, in parks, or semi-public areas (i.e., little niches in the urban public domain). In many countries, both developed and developing, children are never an important target group in urban planning. There are many explanations for the oversight caused mainly by contradictory attitudes towards children’s play that range from a waste of time to a mechanism to benefit learning and development [13]. A further marginalization of outdoor play space in urban planning occurred with increasing influence of private developers in the building process and growing pressure to build in high densities in cities where vacant land in the public domain is scarce and expensive [14]. There are also rising parental concerns for children playing outdoor in an urban area because of fears of social dangers, traffic risks, and safety of play equipment [15].

Standards for the construction and provision of play spaces (including parks, playgrounds, open space, or recreation facilities) in an urban setting vary from place to place. Aside from the issue of quality, the primary concern of a play space is its accessibility measured in terms of the proximity between home and play space or the percent of population served, including those with limited mobility. For example, the US National Recreation and Park Association [16] indicates that 7 in 10 Americans can walk to a play space that has at least one accessible route for people with disability. The UK National Playing Field Association [17] states that a play space for very young, early school age, or older children should be accessible within 1-, 5-, or 15-min walking distance respectively (corresponding to 100, 300, 600 m respectively). Furthermore, 0.8 hectares of children’s play space is needed for every 1000 people, regardless of disability. There are also other factors of consideration besides accessibility, including landscape diversity, facilities affordability, and activities provided in relation to site usability and popularity [18,19].

In a densely populated megacity like Hong Kong (HK) where lands in small inner-city centers are in considerable demand, every bit of space counts. This shortage of urban land has given rise to urban corridors and pocket parks as areas for rest and relaxation. Many of these play spaces or recreation areas are not planned but ‘leftover’ areas interspersed amongst planned and larger constructions. However, the HK 2030+ planning aspiration advocates to improve children’s play needs and the emotional wellbeing of the population [20]. It establishes new standards for home separation distances to open space (within 400 m) and country parks (within 3 km). It also recommends a clear open space standard of a minimum of 20 ha per 100,000 persons, which computes to 2 m^2^ per person (apportioned as 1 m^2^ per person for district and local open space respectively). However, user groups of secondary school students or teenagers are not considered by these planning recommendations.

Play space in HK is provided by three major organizations, namely Leisure and Cultural Services Department (LCSD), Housing Authority (HA), and Housing Society (HS). Play and recreation facilities for children in public space is primarily provided and managed by the LCSD. The LCSD managed play space accounts for about 70% of the public play space provision. The other 30% is provided in non-LSCD venues, such as in public rental and subsidized ownership housing estates managed by HA and HS. A first step towards estimating potential users of play space is to examine the spatial distribution of children based on census and related statistics. The study examined quantity and quality of play space from the spatial perspective to inform planning directions. It focused exclusively on identifying both indoor and outdoor public play areas (operated by the LCSD, HA, and HS) that provide open access to children and where parental or adult supervision is necessary in their use. It would not consider play areas operated by private companies or private housing estates and schools. Despite the deficiency in play space accounting, this compiled data set will form the basis for further spatial analysis of sufficiency of play space at the district level by considering children population and their geographic distribution.

## 2. Methods 

The present study employs a three-stage multilevel mixed-method design to assess the sufficiency of play space in an ultra-dense city. It engages a geographic information system (GIS) and responses from a survey questionnaire to accomplish the following tasks: (i) compile a comprehensive inventory of existing play space; (ii) understand user perception and needs; and (iii) evaluate demand and adequacy in the provision of play space. Ethical approval was granted by the Human Research Ethics Committee of the University of Hong Kong (EA1611003).

### 2.1. Study Area

Hong Kong (22°23′ N, 113°40′ E) is situated in southern China beneath the Pearl River Delta. It measures 1104.41 km^2^ and comprises four major geographic regions of Hong Kong Island (HKI), Kowloon Peninsula (KLN), New Territories West (NTW), and New Territories East (NTE). With around 40 percent of its land still covered by country parks and a population of over 7 million people concentrated in limited amounts of flat land [21], HK is one of the most densely populated cities in the world.

### 2.2. Study Design

#### 2.2.1. Data

A GIS inventory was built to empower spatial analysis of existing play space in HK. Key attributes included location, size, and type of play space, as well as 2016 socio-demographic data by 18 districts of HK available from the Census and Statistics Department [21]. Data about existing play space of HK came from the LCSD. The study used official data from the Survey and Mapping Office [22] to construct a base map of HK including: (i) digital topographic map series (B5000 and B10000), (ii) digital orthophotos (DOP 5000), (iii) building data (BG1000), and (iv) road centerline files (RG1000) for 2011. It also consulted the following data to determine various urban and planned developments in the study area: (v) SPOT-5 panchromatic image of 2.5-m resolution, (vi) multispectral image of 10-m resolution, and (vii) outline zoning map series from the HK Planning Department. 

The study administered a questionnaire survey to obtain data about perception and user needs of play space. The questions focused mainly on visitation habits, attitudes, and play preferences. A small sample of 175 participants, comprising parents of children attending special education schools (6 to 15 years old inclusive) [23], were recruited by convenient sampling method. The surveys were conducted in group-based settings, where adults and their children would complete the questionnaire together as a family. The involvement of adults was important because their opinions towards play and playgrounds directly affect whether or not their children have the opportunity to play in the playground. Although these participants cannot be considered representative of all adults and children (both handicapped and non-handicapped) who use playgrounds, they can provide some understanding of the life experiences and aspirations of specific playground users. Nevertheless, the insights conveyed through this study need to be interpreted with care.

All data processing and statistical analysis were carried out using Microsoft Excel (2010; Microsoft Corporation, Redmond, WA, USA) and SPSS version 24 (2016; IBM Corporation, Armonk, NY, USA). The study employed ArcGIS 10.2.1 (2011; ESRI, Redlands, CA, USA) to process digital map data and conduct spatial analysis. 

#### 2.2.2. Data Analysis

The questionnaire contains 47 questions to address 5 key attributes, including (a) playground usage, (b) accessibility, (c) quantity, (d) quality, and (e) user characteristics. Surveyed responses were summarized using simple statistics to derive user perception in specific locations. The spatial distribution of play space managed by LCSD, HA, and HS was mapped and superimposed over populated areas. These findings were compared against socio-demographic data to evaluate demand and adequacy of play space at the district and region levels. Site visits to selected playgrounds were integrated with GIS analysis to offer qualitative assessments of accessibility and environmental issues. Proximity effects of roads on playgrounds were evaluated by applying GIS-based multicriteria analysis [24] to assess location suitability of playgrounds.

### 2.3. Ethical Approval and Consent to Participate

Ethical approval was granted by the Human Research Ethics Committee of the University of Hong Kong (EA1611003).

## 3. Results

### 3.1. Existing Provision of Play Space

There is currently no comprehensive inventory of play space provision in HK. Information about the play space (size or area, carrying capacity, and target age groups) and play equipment (key attraction, number, and variety) are incomplete for both outdoor and indoor facilities. The LCSD managed 634 outdoor and 34 indoor play spaces in 2016. The size and capacity of LCSD managed outdoor play spaces can only be roughly generalized as set out in Table 1. Besides these prescribed play spaces, some play facilities are conveniently installed in other public areas managed by the LCSD such as rest gardens, sitting out areas, and roadside garden plots. These ‘non-conventional’ play spaces are scattered and normally much smaller in size than an actual playground or park. The quantity and variety of play equipment are also very limited. Records of non-LCSD play space managed by individual housing estates are also incomplete. Table 2 shows that 188 of the 190 public rental housing estates managed by either HA or HS have at least one play space (designated as garden, resting area, or podium) within their compound. It is particularly difficult to determine the size of play space which is not regulated and may be scattered randomly in multiple open areas within a housing estate. Moreover, some play equipment for children are installed in close proximity to or amidst fitness equipment for adults and the elderly.

**Figure 1:**
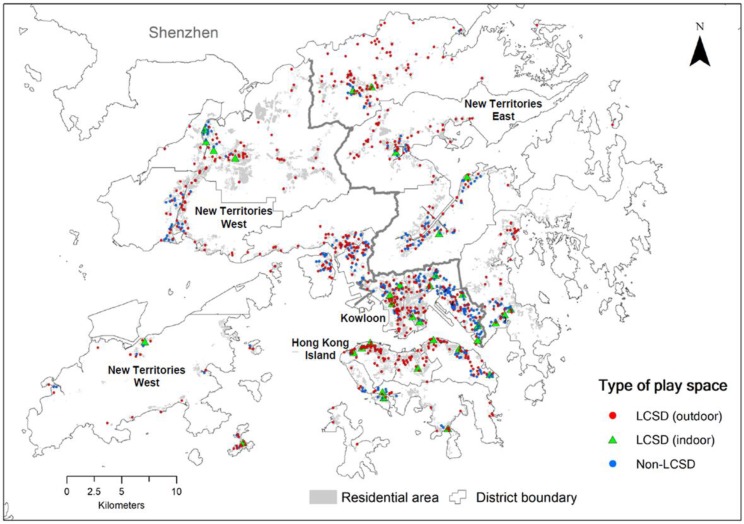
Locations of public play spaces in Hong Kong, 2016.

Figure 1 shows the locations of play space in HK. It can be seen that play areas are generally situated nearby residential areas although some play areas appear to be scattered sparsely in the New Territories within low rise residential developments. Table 3 shows the percentage of residential buildings within 100, 200, and 300 m circular buffers of all playgrounds. These circular buffers may entail walking distances of between 200 m and 600 m because of the winding paths and uneven terrain. The numbers in Table 3 confirm the visual observation that a higher percentage of residential buildings in HKI and KLN are found within 300 m of a playground. These playgrounds are deemed accessible by nearby residents.

### 3.2. User Perception and Site Analysis of Play Space 

Children participants aged between 1 and 12 (mean = 6; 65% boys) and parents/guardians aged 25–70 (mean age group = 35–44; 85% female). These children reported one or more forms of disability (29% oral, 18% aural, 2% visual, 12% intellectual, 14% physical, 8% learning, 26% autism, and 33% developmental). Over 55% of adult participants were born in Hong Kong; all but 3% had primary or above education; over 90% were married; 50% were homemakers and 30% had full-time jobs (see Lai [28] for more detailed breakdowns of participant characteristics).

In terms of visitation habits, 55% used the playgrounds at least once a week, with 15% for ≥4 times weekly. 75% could walk to a playground in less than 20 min. The most popular times of play were evening hours for 30–60 min per visit. Playgrounds were selected because they were accessible (57%), had play components (45%), were safe (43%), and clean or well maintained (35%). In total, 90% of users engaged equipment in the playgrounds where slide (72%) and swing (53%) emerged as the favorite equipment. The majority of parents/guardians felt that playing in the playground was both essential and a preferred leisure activity for their children (mean score = 1.7 out of 5 where 1 is strongly agree). They also rated having a playground near home as quite important (mean score = 8.9 out of 10 where 10 is very important). There was general consensus that playgrounds were of sufficient capacity (crowdedness level was 53% acceptable and 32% high). 

**Figure 2:**
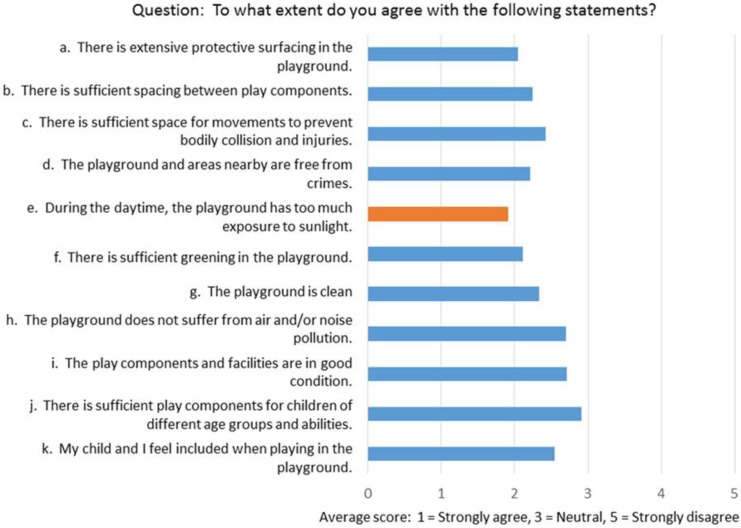
Perception of parents toward playgrounds in their respective neighborhoods.

Results of the questionnaire survey based on a 5-point Likert scale show that parents were generally pleased with the provision of play space and the play equipment (Figure 2) except for the negative comment on too much sun exposure due to a lack of tree cover or overhead protection. Although there was no obvious negative comment about disabled access, many parents/guardians (15–35%) were not aware of amenities for people with disabilities (such as ramp, stair lift, tactile guide path, braille, etc.). They were rather positive about playgrounds being accessible to children with varying abilities (mean score = 2.2 out of 5 where 1 is strongly agree). However, environmental audits of access trails between selected playgrounds and nearby housing estates reveal major problems needing attention. 

**Figure 3:**
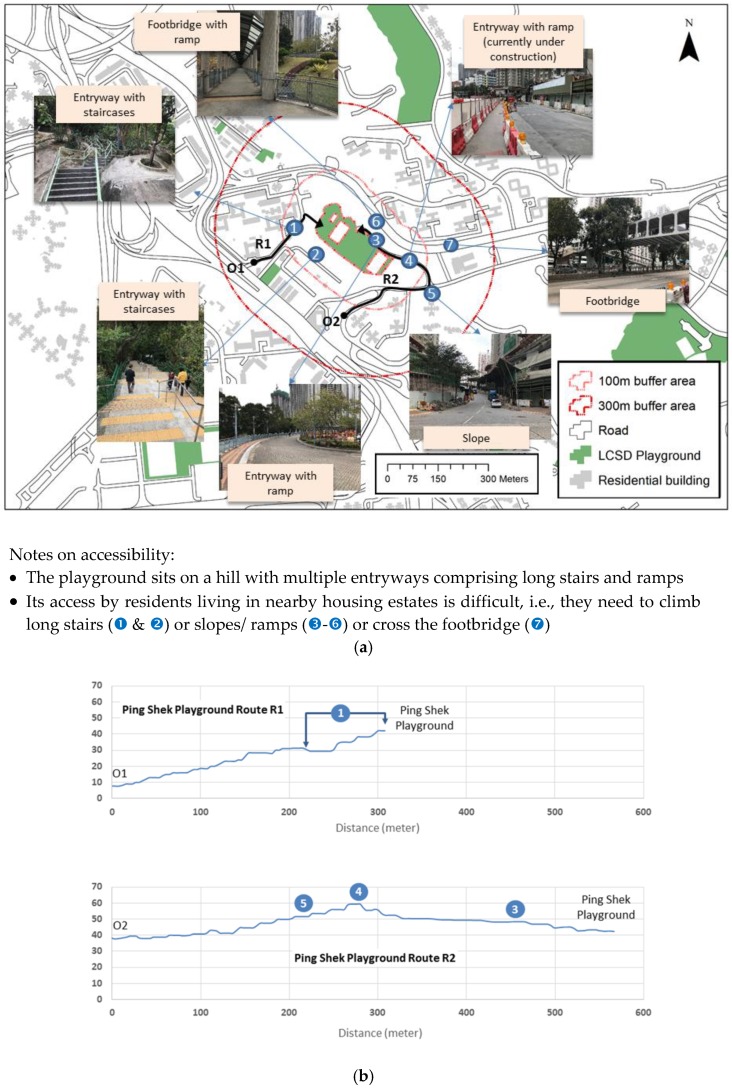
(**a**) Accessibility issues of the Ping Shek Playground in Kwun Tong District, 2016. (**b**) Vertical profiles of routes R1 and R2 to Ping Shek Playground in Kwun Tong District, 2016.

Figure 3a shows an enlarged area of the Kwun Tong district centering on the Ping Shek Playground to reveal two issues. Firstly, there are residential buildings currently not within the 100 m to 300 m buffer areas of the Ping Shek Playground (shown as red boundaries) although they are served by smaller neighborhood playgrounds (shaded in green). Notwithstanding the fact that some buildings are private housing estates (not covered in the present study) that may have their own play facilities to meet needs of their residents, there remains a number of residential properties located beyond easy access to the LCSD and non-LCSD play areas. Secondly, access routes to the playground as indicated by R1 and R2 in Figure 3a represent the horizontal separation without considering the vertical dimension. Cross-section or vertical profiles of the routes shown in Figure 3b give the real perspective of the access. Although shorter in terms of the horizontal distance, route R1 actually requires a steeper climb and a long flight of stairs (labelled as 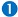 in both Figure 3a,b). The presence of stairs along R1 means that the route is not suitable for wheelchair access even though it lies within the 100 m to 300 m buffer areas of the playground.

### 3.3. Spatial Analyses of Current Locations of Play Space

**Figure 4:**
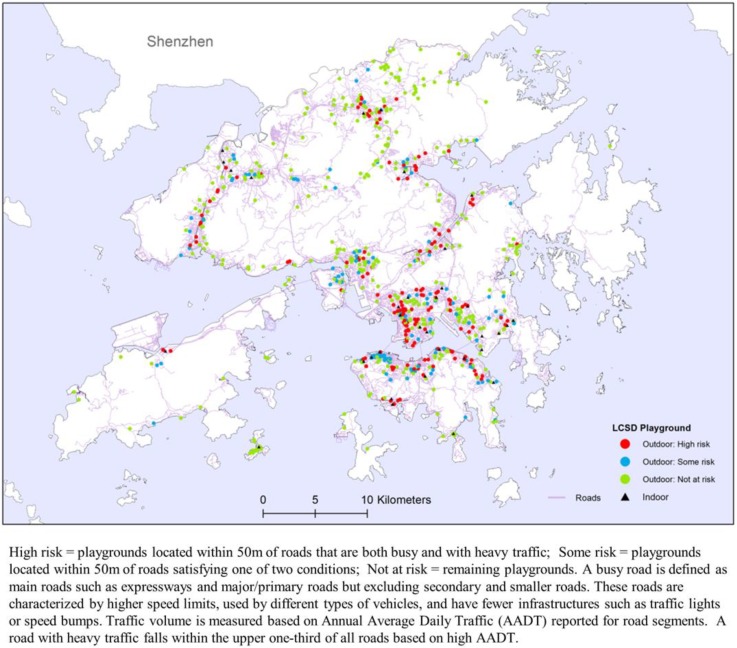
Playgrounds by risk levels by districts and regions of Hong Kong, 2016. Note: Risk in this case involved only two aspects: traffic-related safety and pollution exposure. Other aspects of the playgrounds (such as greening and tree coverage, natural ventilation, risk of injury, maintenance standard, surface impact attenuation, age-specific play equipment, etc.) were not considered. Site inspection of these playgrounds is necessary to examine whether or not the playgrounds are posing both safety and pollution risks. For example, a sunken or fenced playground within a housing estate nearby a busy and polluted main road may pose pollution but not safety risk. Also, a playground in a semi-enclosed canyon setting surrounded by tall buildings has poor natural ventilation leading to more pollutants being trapped. However, a roadside playground of a decent size with sufficient greening and tree cover can reduce air pollution levels. Such location-specific environmental information can only be confirmed by conducting site visits.

An important part of the research is to evaluate the suitability of current location of playgrounds. Figure 4 shows outdoor playgrounds in three risk classes based on two criteria: (i) less than 50 m from major roads, and (ii) less than 50 m from roads with heavy traffic. An outdoor playground fulfilling both criteria is regarded as ‘high risk’; that satisfying either one of two criteria is considered of ‘some risk’; and the remaining is ‘not at risk’. Table 4 shows that about one-quarter (24.1%) of the current playgrounds are positioned in less than desirable locations as they pose safety and health risks. However, there is no significant association between percent risk levels of playgrounds and geographic regions.

**Figure 5:**
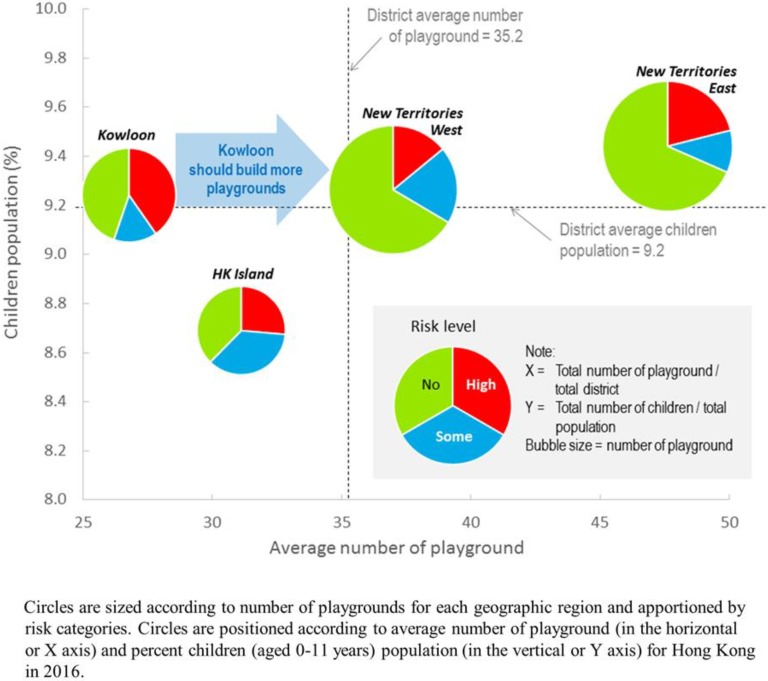
Playgrounds by risk levels by regions of Hong Kong against average number of playground and percent children population, 2016.

A summary of the current situation of play space provision in HK is shown in Table 4 and Figure 5. Although geographic association of percent risk levels of playgrounds cannot be established statistically (Table 4), Figure 5 clearly shows that KLN with a high percentage of children population similar to NTW and NTE (above the district average shown by the horizontal dashed line) has the lowest average provision of play space. The situation is exacerbated by the fact that a disproportionately large percentage of the limited play space in KLN is inferior in terms of safety and pollution standards. In comparison with HKI with the lowest percentage of children population, the number of outdoor playgrounds in KLN is lacking far behind. There is an urgent need to not only increase the provision of play space in KLN but also improve its locational quality.

## 4. Discussion

The WHO recommended minimum open space standard is 9 m^2^ per person within a 15-min walk from home [29]. The current practice of 2 m^2^ per person of open space (which includes play space) in HK is far behind 5.8–7.6 m^2^ for major Asian cities like Tokyo, Seoul, Shanghai, and Singapore [30]. Figure 1 and Table 1, Table 2 and Table 3 show that playgrounds of HK were reasonably distributed in populated areas in 2016. It was also suggested through subjective perception (Figure 2) that users were generally satisfied with the service provision. However, environmental audit at a few selected sites, as illustrated in Figure 3, suggested possible lack of consideration for disabled access. Notwithstanding the commitment of the HK government in enhancing play space, the right of children with disabilities to play is not being fulfilled as a result of the insufficiency of inclusive playgrounds. Except for the inclusive playground in Tuen Mun [31], local playground facilities have been criticized to be all of the same pattern, not diversified enough, and uninteresting.

More creative approaches and designating play space in future land use development as opposed to passive planning is needed to improve the situation. For example, alternative play spaces are needed to improve play conditions while awaiting planning policies to adapt or develop. Studies have shown that children are highly creative in finding ways and alternative places to have fun, such as vacant lots [32]. There is also heightened awareness that healthy child development should involve reasonable and meaningful risk-taking by children [33]. In HK, safety of a play space is a primary if not utmost concern and it is also unlikely that alternative or vacant lots are available given its compact settlement pattern. A possible solution besides creating indoor place spaces is to build elevated playgrounds and public green spaces in intermediate levels or roof-tops of high-rise buildings. 

The use of GIS methodology has enabled considerations of factors not normally included in the evaluation of play space. Existing provision of play space can be analyzed against potential demand at the district and region levels to identify mismatches in different dimensions of need and risk. The criteria for defining need and risk in this study recognize that a variety of factors can influence children’s environmental affordances and these factors can change over time as children grow and develop. In this study, a playground is considered safer if it is situated away from major roads (i.e., highways and primary roads) and its air quality better if it is located away from roads based on high annual average daily traffic (AADT) counts. Although children’s knowledge of the world and their ability to act accordingly have been shown to be conditioned by distance [34], the qualitative distinction between relations of near and far has remained unclear. Here, 50 m was selected as the separation threshold between a playground and other urban features (such as roads or land use types) given the very compact city configuration of HK whereas 300 m (equivalent to 15–30 min of walking) was used as the accessibility threshold between playground and home locations. These distance thresholds are informed by research/practice and they can be adjusted easily in a GIS setting pursuant to changing circumstances. 

## 5. Conclusions

Planning is largely a practice guided by rational scientific approach but with little consideration to cognitive, experiential, and emotional aspects [35]. The loss of public spaces in contemporary urban planning, in particular the public realm for children, has been documented [36,37]. With the more traditional landscapes replaced by spaces for commercialization and development, people’s emotional attachment to places is likely marginalized and weakened. A major challenge in considering issues of urban development and community-building is to cultivate a sense of place and belonging to empower civic responsibility for urban sustainability. Consequently, it is critical to develop stronger attachments to places, especially in children, through provision of open space for them to explore independently, to socialize, and to associate identity.

The study is not without its limitation. Our survey participants from convenient sampling represent parents and children with lesser form of disabilities who may have conformed to or become more tolerant of their entitlement over the years. In contrary, an earlier study by Knowles [38] concluded that many children in HK are being excluded from play because facilities in the city are insufficient and not meeting the needs of children at different ages, especially those with disabilities. Our GIS analysis was hampered by not having access to detailed data about size and type of playground [39]. A clear definition of play space similar to that practiced in the United Kingdom^1^ is needed to improve spatial analyses and better assessment of sufficiency or deficiency levels of the service provision.

In our attempt to examine the convenience of play space for children of HK, we note that the living environment is largely a product of planning policies that rarely cater to the real needs of children. It is not sufficient to just meeting benchmark standards of open space, say 2 m^2^ per person. While the size requirements of the services should be given first consideration, the location, and distribution of various services must be considered in whole as opposed to by piecemeal adjustment. When play space provision and actual needs of children cannot be systematically related, inclusive provision is hardly addressable. In this regard, children of HK shall continue to be deprived of their rights to quality play space when natural play space is taken over for buildings, streets, car parks, and motorways and when play space allocation must give way to availability of space, financial consideration, and administrative convenience.

### Footnote

1 The following definitions of play space are extracted from *Guidance for Outdoor Sport and Play: Beyond the Six Acre Standard (England version)* [17]:

(i) Local Areas for Play (LAP)—These are unsupervised small open spaces specifically designed for young children for play activities close to where they live. Although without play equipment, LAPs have characteristics that make the area conducive to children’s play. Such characteristics include ease of access, a relatively level site, informal surveillance and modest provision of landscaping so that play is not inhibited. The National Playing Fields Association (NPFA) considers that LAPs should be within a one-minute walking time of home.
(ii) Local Equipped Area for Play (LEAP)—These are unsupervised play areas that are equipped for children of early school age. While sharing similar characteristics to LAPs, LEAPs feature a range of different types of play equipment. The NPFA considers these should be located within a five-minute walking time of home.
(iii) Neighborhood Equipped Area for Play (NEAP)—These are also unsupervised but they are intended to service a substantial residential area. While sharing similar characteristics to LEAPs, NEAPs feature a significant range of different types of play equipment. It is equipped mainly for older children but with opportunities for play for younger children. The NPFA recommends these should be located within 15 min walking time of home.

## Figures and Tables

**Table 1:** Types of LCSD managed outdoor play space for children.

Size	Nature of play	Genre	Number *
<0.5 ha	Passive	Sitting-out area	65
		Garden	98
<1.0 ha	Active	Playground	343
>1.0 ha	Active + Passive	Park/Recreation ground	105
	Variable	Beach	5
		Promenade	6
Variable	Variable	Others	12
Total			634

* as at December 2016.

**Table 2:** Playgrounds managed by housing estates. The statistics are computed based on data retrieved from the GeoInfo Map and websites of HA and HS #.

Types of Housing Estate	Stakeholder	Estates with Play Area	Total Estates
Rental			
Public Rental	HA	171 (98.8%)	173
Rural Public	HS	3 (100%)	3
Rental	HS	14 (100%)	14
	Subtotal	188 (98.9%)	190
Subsidized ownership			
Housing Ownership	HA *	97 (49.0%)	197
Sandwich Class	HS *	8 (80.0%)	10
	Subtotal	105 (50.7%)	207
TOTAL		293 (73.8%)	397

HA–Hong Kong Housing Authority; HS–Hong Kong Housing Society; * Managed by Owner’s Corporation; # GeoInfo Map [25]; HA [26]; HS [27].

**Table 3:** Proximity analysis of playground coverage by 18 districts of Hong Kong.

District	Residential Building Area by Districts		Residential Building Area Covered by all Playgrounds (for Different Buffer Sizes) by Districts					
			100 m		200 m		300 m	
	km^2^	%	km^2^	%	km^2^	%	km^2^	%
Hong Kong Island								
Central & Western	0.72	5	0.29	40	0.49	68	0.60	83
Eastern	0.74	5	0.30	40	0.53	73	0.66	89
Southern	0.72	5	0.15	21	0.26	36	0.33	46
Wan Chai	0.55	3	0.13	24	0.28	51	0.38	69
Kowloon								
Kowloon City	0.99	6	0.27	27	0.61	61	0.84	84
Kwun Tong	0.64	4	0.32	50	0.58	91	0.63	99
Sham Shui Po	0.73	5	0.30	41	0.59	81	0.69	94
Wong Tai Sin	0.42	3	0.20	47	0.37	87	0.41	97
Yau Tsim Mong	0.70	4	0.27	38	0.52	74	0.64	92
New Territories West								
Islands	0.76	5	0.15	19	0.30	40	0.40	53
Kwai Tsing	0.54	3	0.28	53	0.47	88	0.53	98
Tsuen Wan	0.52	3	0.19	36	0.35	67	0.45	86
Tuen Mun	1.05	7	0.26	25	0.56	54	0.78	75
Yuen Long	2.59	16	0.31	12	0.66	25	0.98	38
New Territories East								
North	0.98	6	0.36	37	0.63	64	0.75	76
Sai Kung	1.11	7	0.20	18	0.39	35	0.50	45
Sha Tin	1.08	7	0.29	27	0.59	54	0.77	71
Tai Po	1.22	8	0.24	20	0.49	41	0.65	54
**Total**	**16.06**	**100**	**4.50**	**-**	**8.68**	**-**	**10.99**	**-**

**Table 4:** Population distribution and risk levels of playgrounds by four regions of Hong Kong, 2016.

Regions	Percent (%) Children population	Average ^1^ Number of Playground		Percent (%) Risk Levels ^2^			
				High Risk	Some Risk	Not at Risk	Total
Hong Kong Island	8.7	31.3		5.2	7.1	7.4	19.7
Kowloon	9.3	26.8		8.5	3.2	9.5	21.1
New Territories West	9.3	37.0		4.1	5.7	19.4	29.2
New Territories East	9.4	47.5		6.3	3.2	20.5	30.0
Total				24.1	19.1	56.8	100.0

^1^ Average number of playground disregards size factor. ^2^ Chi-square *p*-value = 0.09199 indicates no association between percent risk levels and geographic regions.
